# Dental professional recognition of dental anxiety from a patient perspective: a grounded theory study

**DOI:** 10.2340/aos.v83.42447

**Published:** 2024-12-18

**Authors:** Markus Höglund, Inger Wårdh, Shervin Shahnavaz, Carina Berterö

**Affiliations:** a Center for Orofacial Medicine, Public Dental Service Östergötland, Linköping, Sweden; b Department of Dental Medicine, Karolinska Institutet, Huddinge, Sweden; c Academic Center of Geriatric Dentistry, Stockholm, Sweden; d Department of Health Sciences, University of Karlstad, Karlstad, Sweden; e Center for Psychiatry Research, Department of Clinical Neuroscience, Karolinska Institutet, Solna, Sweden; Stockholm Health Care Services, Region Stockholm, Stockholm, Sweden; f Division of Nursing Sciences and Reproductive Health, Department of Health, Medicine and Caring Sciences, Linköping University, Linköping, Sweden

**Keywords:** Dental anxiety, dental staff, identification, patients perspective, qualitative study

## Abstract

**Objective:**

To explore patients’ experiences of dental staff recognising their dental anxiety.

**Material and Methods:**

Semi-structured interviews were conducted with 10 adults who identified themselves as dentally anxious. The sampling of study participants was purposive, and the audio-recorded interviews were transcribed verbatim. Classical grounded theory and constant comparative analysis were used to analyse the interview data inductively. Theoretical saturation was reached after eight interviews.

**Results:**

*Hope for ‘Fingerspitzengefühl’ from the dental staff* emerged as the core category, which means having an intuitive instinct about a situation and knowing how to react to it. It also is tact or sensitivity that comes with experience. The foundation for this core was four categories: *Dental anxiety causes involuntary signals, Strategies to minimise contact, Seen and respected by the staff, and Recognisable strategies*. The interviewees hoped that the dental staff would notice their dental anxiety and understand their feelings of shame, and take relevant actions to alleviate their anxiety.

**Conclusions:**

Dentally anxious patients often struggle to express their anxiety but hope to find a dentist with *Fingerspitzengefühl*. The result cannot be generalised but transferred to a similar population.

## Introduction

The prevalence of dental anxiety is difficult to determine due to the many different ways to measure it, but it is a well-known problem [1] and the impact on the patients with severe dental anxiety can be profound [2]. Several methods and techniques have been suggested to alleviate dental anxiety [3], but studies have shown that dental clinicians lack the ability to identify high levels of dental anxiety [4–7]. How dental anxiety is recognised has been explored by Höglund et al. using Grounded Theory (GT) [8]. It was discovered that dental clinicians had a relatively homogenous view of how dental anxiety manifests and did not always believe patients who state their anxiety without physical signs or problems during treatment [8]. However, that study only accounted for the perspective of the dental staff. There is a knowledge gap when it comes to the perspective of the patients suffering from dental anxiety. Thus, this article aims to explore patients’ experiences of dental staff recognising their dental anxiety.

## Material and method

Classic GT [9,10] was used as it is suitable to study interactions and social processes based on symbolic interactionism [10]. The method can systematically develop a theoretical construct without a preconceived hypothesis to answer the research question inductively. The theoretical construct is grounded in the data by conceptual categories and the identification of a core category. The core category aims to be the primary answer to the research question. To identify different perspectives in the data, the present research team consisted of dentists experienced in dental anxiety, a psychologist, and a methodological expert [11]. Analysis was primarily performed by the first author, who was guided and supervised by the last author (familiar with GT). The other authors were consulted regularly throughout the data analysis and writing of the manuscript.

Ethical approval was obtained from the Swedish Ethical Review Authority before the study started (Ref. no.: 2020-04633), and conforms to the Declaration of Helsinki [12]. The main ethical concern was to guarantee the confidentiality of the participants. The transcribed interviews were coded with a number, and unique personal information was left out to ensure that no personal information could be identified. A research protocol was the foundation for the ethical approval and guided the research process. This protocol was not registered elsewhere.

The results were reported following the Consolidated Criteria for Reporting Qualitative Studies (COREQ) guidelines [13], which are supplemented by a supplementary file.

### Setting

The study participants were adults identifying themselves as suffering from dental anxiety. A snowball recruitment was planned. The initial recruitment was of informants known by the first author to suffer from dental anxiety, but as none of these knew anyone else suffering from dental anxiety, a convenience and purposeful sample was instead chosen, recruited from the research team’s professionals’ circles by asking colleagues from different dental clinics if they could be helpful with contacts. Inclusion criteria were to be an adult suffering from dental anxiety or having previously suffered from dental anxiety. We excluded anyone with experience of working in a dental setting, as their answers could be influenced by their professional perspective. The first contact was by e-mail; if the person showed preliminary interest, a formal invitation was sent. The participants were given verbal and written information about the aim and method, and could ask questions about the study. They were allowed to freely choose the interview method (in-person meeting, digital video meeting, or by telephone). All participation was voluntary and they could at any moment withdraw from the study without negative consequences. Upon acceptance, written consent was obtained, and the level of dental anxiety was estimated using the Modified Dental Anxiety Scale [14]. A total of 10 adults participated, 5 females and 5 males aged 37–68 years. The interviews were between 33 and 64 minutes with an average of 46.5 minutes. Six interviews were done face to face, of which four were performed in the interviewee’s home, one at the interviewee’s job, and one in the interviewer’s home. Two interviews were done via Skype and two by telephone; three interviewee’s were at home during the interviews and one was at work. The interviewee’s MDAS score varied between 9 and 22, averaging 13.4.

### Data collection

No one but the interviewer and the interviewee were present during the interviews that all started with the same question: ‘Please tell me about your last visit to a dental clinic’ to make the interviewees more comfortable with the situation. The central question was: ‘How can dental staff become aware of your dental anxiety?’ The research team had constructed a semi-structured interview guide to assist the interviewer. It evolved and grew with new questions after each interview, as the need of further data were discovered. For example, when reluctance to reveal dental anxiety to the dental staff appeared, this question was added to the interview guide. Other questions added included issues about mood and anxiety affecting interaction with dental personnel. Adding new questions is a correct mode in GT to get more information to fill the categories, and reach saturation [10].

The first author, a trained and experienced interviewer, conducted all the interviews in a conversational style, listened actively, and used probes like ‘can you tell me more about…’, ‘you said earlier that…, can you elaborate on that’ to extract as much data as possible. If the interviewer found a subject interesting, even one not present in the semi-structured interview guide, he was free to explore this. Field notes were taken after each interview and during the data analysis.

### Data analysis

The interviews were audio-recorded and transcribed verbatim by the first author and feedback was provided by the last author after each interview. The first and last authors performed most of the analysis; the other authors were informed after each interview and provided input when they deemed it necessary or when asked by the first author. The transcript was analysed and coded according to the steps suggested in GT [9]. Analysis was performed concurrently with the data collection and continued until theoretical saturation was reached (after eight interviews), followed by two more interviews conducted to certain that theoretical saturation had been reached.

The transcripts were analysed line by line and open coding was used to answer the question ‘How can dental staff become aware of your dental anxiety?’ Data were arranged into groups of substantive codes and data that fit together were formed into categories. After each interview, codes and categories were analysed, compared, rewritten, and rearranged. There were continuous discussions, and consensus was achieved about the meaning of the data as the categories and the core category developed [11]. Selective coding was used to elaborate the categories and their relationships with one another and the core category [9]. Categories were considered saturated when data did not add new information. After the final data collection, the theoretical construct or ‘core category’, providing the primary answer to the research question was established.

The first data appeared heterogenic. For example, one interviewee recalled fainting in the dental chair but stated only a few sentences later that her dental anxiety was probably hard to spot. All interviewees wanted the dentist to know about their dental anxiety but several stated that they were reluctant to share this information due to past experiences of being met with disbelief. However, as the cycle of interviews and analysis continued, a pattern emerged. Around interview six, the categories (Dental anxiety causes involuntary signals, Strategies to minimise contact, Seen and respected by the staff, and Recognisable strategies) had emerged as distinct entities without overlap, and around interview seven, the core category of ‘Fingerspitzengefühl’ was established.

### Validity/relevance check of result

Finally, the first author (interviewer) presented the result by telephone to two of the interviewees. They were encouraged to give their opinions on the study conclusion and to provide feedback.

## Results

The core category was identified as hope for ‘Fingerspitzengefühl’ from the dental staff, based on four categories: Dental anxiety causes involuntary signals, Strategies to minimise contact, Seen and respected by the staff, and Recognisable strategies. The results section first presents the core category and then the categories. All categories are at an abstract level, while the quotations presented are at a descriptive level. In classical GT, the result often is presented without quotations, but here some are added as a service to the reader.

### Hope for ‘Fingerspitzengefühl’ from the dental staff

We define ‘Fingerspitzengefühl’ as a combination of tact, diplomacy, and a certain amount of sensitivity to the feelings of others. It is a quality that can enable a person to ‘negotiate tricky social situations’ [15]. The interviewees felt that the dental staff needed several skills to identify the patients suffering from dental anxiety: a high level of awareness and intuition. However, this was not enough. In a very tactful way, the dental staff must do the right things, listen, and ask the right questions to build trust, enabling the dentally anxious patient to share their burden of dental anxiety with the dental staff. The right thing to say and do varied among the interviewees, and thus the dental staff required ‘Fingerspitzengefühl’ to navigate these tricky situations Although ‘Fingerspitzengefühl’ seems intuitive, experience is essential and effort is required to convey to the anxious patient that the dental staff are aware of their situation. All the interviewees wanted their dentist to know that they suffered from dental anxiety. However, due to feelings of guilt/shame and not wanting to show vulnerability some of the interviewees were reluctant to share this information and others actively tried to hide their dental anxiety from the staff.

‘It is embarrassing and shameful that a grown man does not dare go to the dentist.’ (Interview C)

Most interviewees believed that their dental anxiety was hard to notice. When asked why, their conclusions were based on past experiences where they felt that the dental staff did not notice their dental anxiety or did not appropriately respond when they stated their dental anxiety. Some meant that the dental staff, either missed the signals or did not take it seriously. Thus, they concluded that noticing their dental anxiety required an exceptionally skilled or caring dentist. Most participants had established a relationship where they were confident that their current dentist was aware of their dental anxiety:

‘I get the feeling that they know I am afraid.’ (Interview I)

Although several were still reluctant to talk about their dental anxiety with their dentist arguing that they do not want to burden any other person with their problems.

The staff must have ‘Fingerspitzengefühl’ to sense and act on the patient’s needs, enabling the trust necessary for patients suffering from dental anxiety to confide their problem.

### Dental anxiety causes involuntary signals.

The interviewees know of these signals based on observations or feedback from the dental staff. Several of the signals were closely associated with stress-related activation of the sympathetic system, such as sweating, holding their breath, increased pain sensitivity, hyperventilation, pale face, fainting, and flight response.

Several other signals were to a lesser extent associated with the sympathetic system, for example, involuntary movements of limbs or crying and involuntary sounds like eeEEEEeeE:

‘I have a bit of difficulty being completely still and relaxed, maybe like waltzing around a bit [in the dental chair].’ (Interview E)

Some said that they were highly talkative due to their anxiety, showing nervousness with ‘oral diarrhoea’. Others would be silent and reticent at the dentist.

There were also involuntary changes in the interviewees’ mode. Being less sociable or somewhat depressed. Some expressed a feeling of being absent:

‘You seem slightly absent-minded like that, your thoughts are on something else … I get bitter and locked in and I do not want anyone to talk to me.’ (Interview F)

The interviewees did not choose their mode of behaviour, instead they felt that it was involuntary caused by the impending dental treatment, and outside their control.

### Strategies to minimise contact

Some interviewees had avoided the dentist for 10 years or more and only sought treatment when strictly necessary, such as when experiencing toothache. Others managed to go routinely but had a low threshold for cancelling, almost actively seeking a reason for cancellation. Similarly, to minimise their time at the dentist and exposure to a highly unpleasant topic, several of the interviewees avoided discussing their dental anxiety with the dental staff:

‘You want to avoid talking about things you do not like … then you do not want to bring it up.’ (Interview A)

Some minimised their exposure by requesting sedation to achieve complete or partial amnesia, and surrendered their autonomy and all decision-making to the dentist. Their solution was not to think any more than necessary about their teeth and dental treatments:

‘Do what you need to do, I lie down here and open my mouth and then you get to dig.’ (Interview B)

Some lessened their long-term need for dental treatment by high levels of self-care and proactively seeking the dentist for minor issues to prevent dental problems from becoming more problematic. Another strategy to minimise exposure to dental settings was being a highly cooperative patient, thereby minimising time spent at the dentist. The more adaptive and still, the faster they hoped their dental treatment would go.

Compared to the category of involuntary signs, in this category the interviewees were more focussed on their own experience, and it may be more challenging for the interviewees to consider this category from the dental staff’s perspective. Another possible explanation is that they had received less feedback from the staff on these types of behaviours. Some interviewees thought that the dental staff might pick up on frequent cancellations and rescheduling. A request from the patient for sedation usually comes with a reason as to why they want to be sedated. Hopefully, a dialog between dentists and patients occurs establishing an agreement on what the challenge requiring sedation is. The interviewees did not explicitly disclose if they thought being highly cooperative or quickly leaving the dental office, were things the staff might notice.

### Seen and respected by the staff

To be seen and respected was of great importance to the interviewees. They stated that their dental anxiety brought feelings of guilt/shame and to be a burden for the dental staff.

‘You kind of do not want to put that burden on someone else, you keep it to yourself.’ (Interview D)

These emotions prevented several interviewees from disclosing their dental anxiety to the dental staff. They found it easier to share if the staff were calm and sensitive, as it made them feel seen and accepted. The staff’s respect and reassurance that their dental anxiety was not a problem were equally important:

‘I think it good that they know and … that I can show [that I am anxious].’ (Interview J)

The interviewees felt respected when the staff acknowledged and followed their requests regarding the treatment and were attentive which underscores the importance of the audience’s role in patient care. Several interviewees wanted the dental staff to actively ask about dental anxiety, but preferably indirectly to get an honest answer due to feelings of shame. All interviewees could give multiple examples of when they felt their former dental staff did not see or respect them:

‘The gap between how I experience dental care and how someone who is not dental-fearful experiences it, is so huge, like that gap, so you do not understand. Maybe [the staff] do not understand because if you work in a dental practice then you think it is the most natural thing in the whole world.’ (Interview B)

The interviewees experienced extreme disrespect when dental staff questioned their experience or belittled it while commenting that the treatment did not hurt. When this happened, the interviewees were more reluctant to confide their dental anxiety. Usually, these unavailing attempts resulted in the interviewees seeking a different dental clinic. Furthermore, several interviewees did not like when there was no time to talk about their experiences after the treatment, only the bare minimum to perform the treatment:

‘They just administer, there is not the same … feeling that there is time to talk, they just work.’ (Interview G)

Patients suffering from dental anxiety have an extended need to talk, perhaps more so than regular non-anxious patients. The dental staff’s failure to acknowledge dental anxiety increased the interviewees’ unease with talking about and signalling their feelings.

The interviewees generally thought that their dental anxiety was hard to spot since it was mostly something they felt and could only be addressed if the staff were highly perceptive:

‘If you are a bit sensitive as a practitioner of the profession if I were to enter a dentist’s room then you could probably notice it a little. Then you must have your antenna out or have it in your personality.’ (Interview E)

These statements were often based on the experience that previous dental staff failed to acknowledge their dental anxiety, saying something that gave the impression that they had not taken it into consideration at all. Some of the interviewees were afraid that the staff had forgotten that they suffered from dental anxiety. Sometimes the staff forgot the interviewee’s specific treatment requests despite them being clearly stated.

None of the interviewees said anything negative about their current dental staff and almost all of them felt it was essential to go to the same dentist – being acknowledged is a security. Several of the interviewees had actively developed a personal relationship with their dentist to ensure that the dentist knew about their dental anxiety, but also, to some extent, to alleviate the fear of the dentist:

‘It was very important that you got to know the dentist, it wasn’t just a dentist but there was a person behind… you have … have humanized the dentist …from being a tool for horror and fear to becoming another human who treats you.’ (Interview C)

The category ‘Seen and respected by the staff’ is two-sided; it contains positive experiences of feeling trust in the dentist when dental anxiety was seen and respected, but it also contains examples of the opposite. This category was of great importance to several interviewees, especially if they were to return to the same clinic.

### Recognisable strategies

These strategies, recognisable to the dental staff as signs of dental anxiety, could be divided into three groups. The interviewees actively and voluntarily used them to undergo dental care, not to lower their dental anxiety.

The first group was concepts of distraction and avoidance. The interviewees expressed several ways they might appear distant and absent-minded as they distracted themselves before or during treatment. Prior to treatment, their anxiety could be expressed as nervously fiddling with a pen or their phone:

‘I would have probably sat and read and tried to switch it off by having something to occupy myself with.’ (Interview A)

Another strategy, possibly harder to notice, was to avoid stressful visual stimuli prior to treatment. They did not look at the instruments, just focussed on reaching the chair.

During treatment, the dental staff could discern the patient’s distraction strategies. The interviewees often engaged in small talk. Avoidance of dentistry-related topics was a more overt sign to divert their attention from the dental situation:

‘I try to distract myself by talking about everything else and … get away.’ (Interview H)

Further distraction strategies to disturb the brain, were for example to wiggle in the dental chair, frequently need to clear the throat or request to spit frequently A handful of interviewees actively asked the dental staff to distract them during treatment and not talk about the treatment. This request was a clear signal to the staff that they needed to be mentally occupied with something else than what the dentist was doing.

The second group of tactics focussed on control. The interviewees tried to achieve control by avoiding surprises and always being prepared, paying minute attention to what the staff did and by asking the dentist to inform them extensively about the procedure before treatment. Several interviewees tried to make a verbal contract with the dental staff, dictating what was allowed during the treatment. If the dentist strayed from the verbal agreement, they described this as highly anxiety-provoking:

‘If you have finished fixing a tooth and the dentist says to me, “but you have a small hole there too. While you are here we will fix this” then there will be chaos in my head.’ (Interview F)

The most evident control strategy was one interviewee emphasising to the dental staff that he would decide which treatment would be chosen even if the dentist was the expert. How noticeable the control tactics were to the dental staff varied significantly. Most control tactics require a dialog with the dental staff, but several of the interviewees did not want to mention to the staff that they suffered from dental anxiety; they were worried that they might appear as strange.

The final strategy was to endure and think that this situation is a few minutes of your life; bite the bullet and survive. The interviewees worked mentally to prepare themselves before the treatment and actively used different mental processes to endure the treatment. Perhaps most noticeable was the use of active breathing and controlled relaxation. The enduring strategies were probably challenging to notice by the staff, given that they almost exclusively happened in the interviewee’s mind and were not overtly expressed:

‘I do not think it is noticed much, more than this inner feeling, still, I think it stays inside.’ (Interview D)

### Validity/relevance check of result

The two interviewees who were presented the result recognised the conclusions, and they confirmed that the description of the results was relevant and based on the data they provided.

## Discussion

The core category that answers the research question is ‘Hope for Fingerspitzengefühl from the dental staff’, which means a combination of tact, diplomacy, and a certain amount of sensitivity to the feelings of others. Persons suffering from dental anxiety often lack confidence in their ability to handle the dental situation [4,16]. This aligns with avoidance being a significant issue for those with dental phobia. The dentally anxious, in their struggle, tend to avoid informing the dental staff about their anxiety, instead placing the responsibility of noticing on the dental staff. Understanding this avoidance behaviour is crucial for dental professionals and researchers to better empathise with their patients.

Several of the interviewees had experienced dental treatments where they felt that the dental staff should have noticed, and acted on their anxiety and discomfort but failed to do so. This was often referred to as extremely anxiety-provoking and presents an opportunity for improvement. It may be due to several reasons; perhaps the dental staff did notice the anxiety but failed to signal this to the patient, or perhaps the interviewee was too stressed to pick up on this information from the staff. In a recent study, interviewed dental clinicians stated that they did not believe patients who said they were dentally anxious if they did not show the ‘right’ physiological signs or if problems did not arise during the dental treatment [8]. Furthermore, several studies have suggested that dental staff lacks ability to notice highly dentally anxious patients during routine dental treatments [4–7].

It is not up to the authors to say how anybody should treat a patient suffering from dental anxiety. Previous research [17] as well as psychological literature [18,19], points to a validating attitude, affirmation, and being calm, empathetic, accepting and non-judgemental. These qualities require awareness and perhaps training to use actively and should be given extra focus when interacting with patients suffering from dental anxiety. Motivational interviewing and third-wave cognitive behavioural therapies provide much of what the interviewees in this study asked for. They could be a guide to meet this patient group’s needs but this is outside the scope of this article. It might be an area for further studies.

To the author’s knowledge, there are no previous studies specifically addressing how patients suffering from dental anxiety experience dental staff becoming aware of their dental anxiety. Thus, it is hard to compare the specific findings of this study. However, several of the found concepts share strong similarities with well-known concepts from previous studies, that strength the validity. Shame and guilt is earlier recognised [16,20,21], and this article finds further proof for this. The importance to build trust between the dental staff and the dentally anxious patient is also highlighted in several previous studies [22–25], as are control-seeking behaviours [8,22,24]. Similarities with previously published literature increase the trustworthiness and suggest that our conclusions fit well within the current knowledge in the field. However, the applicability is always up to the reader to decide.

A limitation of the study is that the planned snowball recruitment was abandoned. The chosen purposeful sample may have limited the number of perspectives. Saturation was reached after eight interviews and two more interviews were conducted to validate and secure this saturation, demonstrating the thoroughness of our study. The rather early saturation could be due to the interviewees having similar experiences despite differences in age, gender, education, profession, geographical location, and dental care.

However, we do not know if some perspectives are missing as some people have problems talking about their dental anxiety. An alternative approach could have involved patients diagnosed with dental phobia, potentially yielding additional insights.

To bridge the power gap between the interviewer and the interviewees, the interviewer wore casual clothes during the interviews and informed the interviewees that they were the experts on their perspective. None of the participants were patients of anyone in the research team.

To conclude, this study’s most important finding is the fact that the interviewees want the dental staff to know about their dental anxiety but that dental professionals must be made more aware of the patients’ reluctance to share their experiences. This understanding can significantly affect the dentist-patient relationship and it is likely to impact success of the treatment.

## Supplementary Material

Dental professional recognition of dental anxiety from a patient perspective: a grounded theory study
